# Reconstruction of time-shifted hemodynamic response

**DOI:** 10.1038/s41598-022-17601-5

**Published:** 2022-10-19

**Authors:** Bärbel Herrnberger

**Affiliations:** Department of Psychiatry and Psychotherapy III, Ulm University, Ulm, Germany

**Keywords:** Neuro-vascular interactions, Sensory processing, Visual system, Computational models, Data processing

## Abstract

Regression of voxel time course onto expected response is a standard procedure in functional magnetic resonance imaging that relies on exact onset time and shape of superimposed hemodynamic response functions. Elegant capture of time deviation by time derivative regressors appears complicated by shape distortion and limited to ±1 s, and is usually not exploited for reconstructing the true time-shifted response function together with its magnitude. This analysis of the time-derivative approach provides closed-form functional relations between time shift and regression coefficients that allow for hemodynamic shifts of ±5 s and can explain shape distortion and reconstruction behavior. Reliable absolute latencies were no smaller than 0.6 s in a best-case experiment. Confusions of latency are a previously undiscussed shortcoming where current limitation strategy may eliminate correct latencies and protect incorrect ones.

## Introduction

The hemodynamic response function (HRF) is the impulse response^1^ of the brain to neural activity^2–5^ and key to quantifying the blood oxygen level-dependent (BOLD) signal^6, 7^ from functional magnetic resonance imaging (fMRI). The function describes the temporal evolution of oxygenated hemoglobin in the vasculature, following oxygen consumption by activity-related metabolism.

Established procedure towards maps of task relevant brain areas^8^ regresses measured voxel time courses onto expected superpositions of standard HRFs^2, 9^ whose different onset times are the different stimulus times. Stimulus models other than Dirac impulses require replacement of the HRFs by their convolutions with the specific shape function.

For true BOLD response magnitude to appear in the regression coefficient, and apart from HRF shape variants^10–14^, such procedure must take into account unknown shifts in time that may come from region-specific neurovascular coupling, different stimulus quality or familiarity^15, 16^, reaction time, genetics^17^, age and disease^18^, stress^19^, unknown internally triggered processes, or specification failure of onset time.

Optimization would shift the expected BOLD response by a number of latencies, often determined from gradients of a cost function, until a best match with the measured response. This requires one computationally intensive pseudoinverse for each latency, or each combination of condition-specific latencies, in each voxel. Motivated by Taylor series and with considerably less computational effort, time derivatives^20^ are sensitive to time shift and may abbreviate optimization. They are used within additional regressors, hence estimation would still take only one pseudoinverse for all voxels.

The time derivative is a proper choice even if in regression, unlike the Taylor series, the coefficients to shifted and standard HRF are not the same. Reconstruction of latency and magnitude requires a dedicated processing of the coefficients to the HRF and the derivative; the derivative coefficient alone would only indicate the presence (and the sign) of a response latency. Given orthogonality to both the HRF and the constant regressor, a change in the coefficient to the HRF regressor on introduction of the derivative would solely be due to new correlations with other regressors^15^, never indicate any correction for time shift.

Latency has been retrieved from an asymmetric coefficient ratio–latency function with a latency range of about ±2 s^15^. This function was calibrated on a single shifted HRF and two regressors but on application saw coefficient ratios from complex experimental designs with multiple HRFs and more than two regressors. During calibration, the standard HRF is shifted in time for a number of predetermined known latencies; each shifted HRF is decomposed into standard HRF and time derivative, finally the ratio of derivative to HRF coefficient is assigned a latency. On application, the calibrated function receives a coefficient ratio and returns a latency.

Rather than the known latency of the decomposed HRF, the assigned latency was determined as the difference between maximum positions of the unshifted HRF and a more or less seriously distorted HRF synthesized from the sum of HRF and time derivative, weighted by their estimated coefficients. Distortions were accepted within ±1 s, about the linear range of the ratio–latency function. Respective restrictions have been applied to either the latency, or the coefficients^15, 16, 21, 22^. In contrast, symmetric calibration curves with latency limits beyond ±4 s emerged upon decomposition into basis functions from singular value decomposition and assignment of coefficient ratios to the known shifts in time^23^. Significance of latency has been assessed in groups of subjects, not in the single subject.

Regarding reconstruction of magnitude, addition of the derivative coefficient to the HRF coefficient led to larger or smaller value, depending on shift direction^24^. Such behavior was not reported for an enhanced HRF coefficient, computed as a signed Euclidean norm of the vector of HRF and derivative coefficients, after multiplication by their respective regressor norms^16, 21^. However, lost effect over subject group^25^ by larger subject variance^26^ spoke against such enhancement, same as absent qualitative improvement at restricted latency in favor of retained HRF shape or retained group effect^26^.

The distortions in combined HRF and time derivative remained confusing and unexplained, as a shift in time is not necessarily associated with altered magnitude or shape^27, 28^, and unless they include undistorted shifted HRFs as well, the different shapes would not express HRF variability.

This paper shows that the shape problem arises from attributing the distortions to brain response; it disappears with a change of perspective, from synthesis of a shifted HRF to analysis. From the functional relation of the unknown shift and magnitude to the observable regression coefficients the analysis derives equations for the unknowns that may clarify current procedure, or serve as alternatives. Monte-Carlo simulations then help determine a preferable variant of magnitude retrieval, by looking at bias and shape of distributions. The simulations also validate a proposed variance relation between shift and magnitude, for use in single-subject t-tests of latency. Finally, a best-case experiment investigates if these distributions hold with fMRI data, explores latencies within one sensory domain along with their impact on magnitude, quantifies reliable latency, and illustrates persisting limitations from confusions of latency.

## Results

### Analysis

Regression decomposes a shifted, scaled, and biased function of time *t* as

1 $$\beta \,f(t{+}a) + C = \sum _{i=1}^n \frac{\beta_i(a,\beta ,L_i)}{L_i} f_i(t) + \epsilon (t,a,\beta ).$$

$\beta$ is the unknown magnitude, *a* is the unknown time shift. $a\,{<}\,\mathrm{0}$ shifts to later time, so $-a$ is a positive latency. *C* is a bias. $\beta _i(a,\beta,L_i)$ are the coefficients to the regressors $f_i(t)$, $L_i\,{>}\,\mathrm{0}$ are the known scalars for normalization, and $\epsilon(t,a,\beta )\,{\bot}\,f_i(t)$ for all *i* is an orthogonal deterministic residual function. Specifically, $f(t)\,{=}\,f_1(t)$, $\frac{d}{dt} h(t)\,{=}\,f_2(t)$, and $n\,{=}\,\mathrm{3}$; the last regressor is a constant function, $f_n(t)\,{=}\,\mathrm{1}$.

For the HRF as the difference of two Gamma probability density functions (PDFs) of time^20, 29^,

2 $$f(t) = g(t,p_1,q_1) - c\,g(t,p_2,q_2)$$

3 $$g(t,p_i,q_i) = \frac{{q_i}^{p_i}\,t^{{p_i}-1}\,e^{-{q_i} t}}{\Gamma ({p_i})},\, t\,{>}\,0$$

(where $p_i$ are shapes, $q_i$ are rates, $\Gamma$ is the Gamma function, and *c* is a factor (A23–A26), see supplementary Appendix), and its time derivative (A27), $f_1(t)$ and $f_2(t)$ are mutually orthogonal (A29), same as $f_n(t)$ and $f_2(t)$ (A30). $f_n(t)$ and $f_1(t)$ are correlated (A25), and $\epsilon (t,a,\beta )$ is nonzero for $a\,{\neq}\,\mathrm{0}$. Back to (1), $\beta$ and $\beta _1(a,\beta ,L_1)$ have no unit, and since $\frac{d}{dt} f_1(t)$ is in units of $\mathrm{s}^{-\mathrm{1}}$, $\beta _2(a,\beta ,L_2)$ is in units of seconds.

Solutions to the coefficients $\beta _i(a,\beta ,L_i)$ (A2) are weighted sums of normalized correlation integrals

4 $$k_j(a) = \frac{\int_{-\infty}^\infty f(t{+}a)\,f_j(t)\,d t}{\int_{-\infty}^\infty f_j^2(t)\,dt},\,j\,{=}\,1,\dots,n.$$

The weights (A3) depend on regressor correlations. *C* solely appears in the coefficient to the constant regressor, and by orthogonality (A30) $\beta _2(a,\beta ,L_2)$ (A7) is the same with or without that regressor.

**Figure 1:**
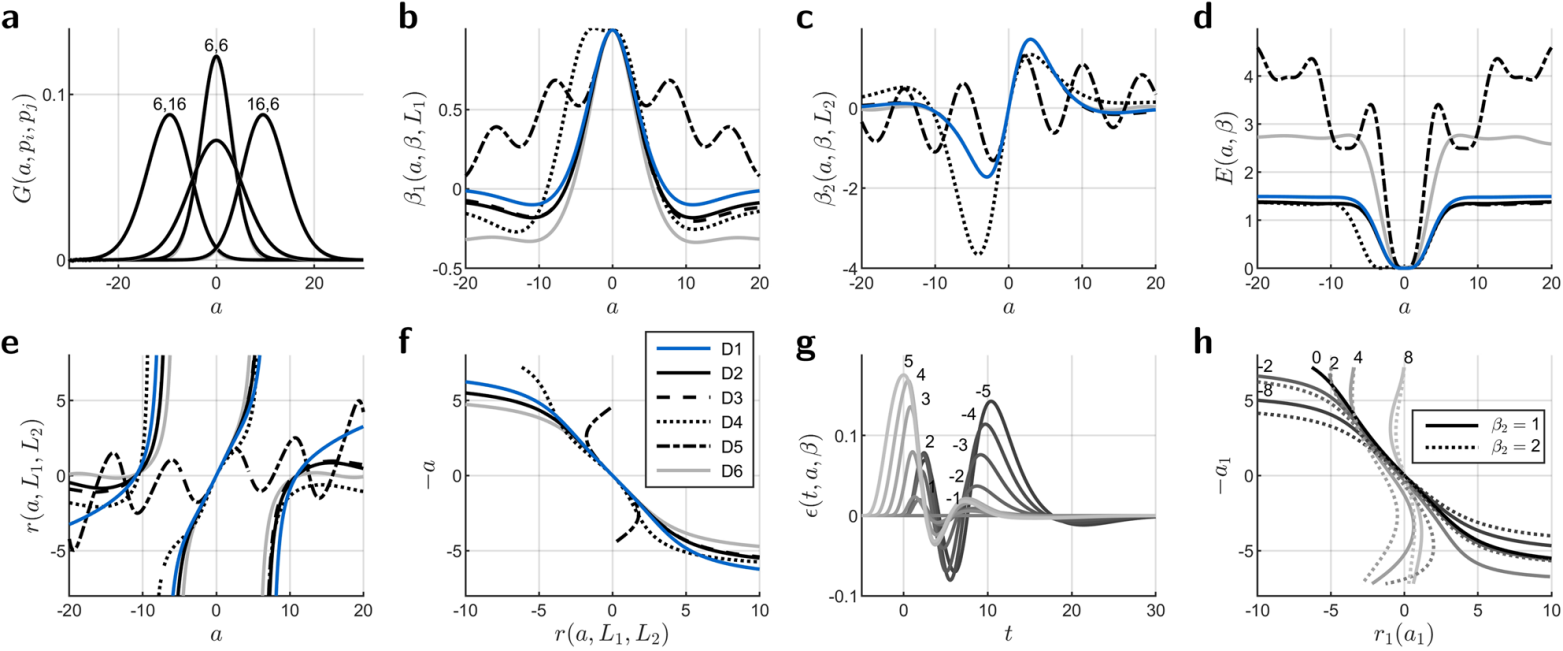
Shift dependencies for parameters of HRF decomposition. Shift *a* is in seconds. Residual functions $\epsilon(t,a,\beta )$ and residual power $E(a,\beta )$ (A1) are in arbitrary units. (**a**) Components $G(a,p_i,p_j)$ (5) for combination (A36) into $k_1(a)$ (4). Numbers indicate shape parameters $p_i$ and $p_j$. When omitted, $p_i\,{=}\,p_j\,{=}\,\mathrm{16}$. All components have practically identical least-squares approximations by single Gaussians or Gamma PDFs, (**b**) HRF coefficient $\beta _1(a,\beta ,L_1)$ (A6), (**c**) Derivative coefficient $\beta _2(a,\beta ,L_2)$ (A7), (**d**) Residual power $E(a,\beta )$ (A1), (**e**) Coefficient ratio $r(a,L_1,L_2)$ (6), (**f**) Central ratio–latency function $-a(r,L_1,L_2)$ from $r(a,L_1,L_2)$ by interchange of axes and interpolation, all for $\beta \,{=}\,\mathrm{1}$ and $L_1\,{=}\,L_2\,{=}\,\mathrm{1}$, (**g**) Examples of residual functions. Numbers indicate shift in seconds, (**h**) Central ratio–latency functions $-a_1(r_1,L_1,L_2)$ for condition $m\,{=}\,\mathrm{1}$ in the presence of another condition $m\,{=}\,\mathrm{2}$. Here, $\beta _m$ is the magnitude in condition *m*, $\beta _1\,{=}\,\mathrm{1}$. Numbers on curves indicate $a_2$ in seconds. Both conditions contain only one HRF. The HRF of condition $m\,{=}\,\mathrm{2}$ is 8 s later in time. The legend in panel (**f**) applies to panels (**b**–**f**). D1–D6 are different versions of decomposition (1). D1, Omission of the constant regressor, D2, Decomposition as is, D3, Regressors and input filtered with SPM highpass filter at 128 s cutoff time (curves mostly overlap with D2), D4, Additional correlated regressor (the non-orthogonalized dispersion derivative), D5, Regressors with 5 HRFs or derivatives 8 s apart. Latency can be retrieved within ±2 s, D6, Regressors with 15 HRFs or derivatives about 32 s apart, filtering as in D3. The curves for D3, D4, and D6 are asymmetric. The latency range for D6 is [−5.51, 5.50], smaller than for D3 because of the multiple HRFs. All curves except D1 and D2 were calibrated.

The functions $k_j(a)$ exist in closed form. Symmetric $k_1(a)$ (A36, A38) is another weighted sum, this time determined by the factor *c* in the HRF (2). The four components

5 $$\begin{aligned} G(a,p_i,p_j) = {} e^{q_j a} & \,\frac{q_i^{p_i}\,q_j^{p_j}}{(q_i{+}q_j)^{p_i{+}p_j}}\,\dots\nonumber\\ & \, \cdot\,\sum _{k=0}^{p_i-1}\,{p_j{+}k{-}1 \atopwithdelims ()k}\,g(a,p_i{-}k,q_i{+}q_j)\,S(a,p_j{+}k,q_i{+}q_j) \end{aligned}$$

(arguments $q_i$ and $q_j$ omitted for simplicity), Fig. 1a, are each built of Gamma PDFs of shift, same as their derivatives (A35). $(i,j)$ is one pair of Gamma PDFs from shifted and standard HRFs, and *S* (A34) flips the function when $a\,{\le}\,0$. Since $k_n(a)$ is a constant function, $\beta _1(a,\beta ,L_1)$ (A6) is symmetrical with respect to $a\,{=}\,\mathrm{0}$, Fig. 1b.

Apart from the sign, the correlation integrals of $f(t{+}a)$ with successive HRF derivatives with respect to time are successive derivatives of $k_1(a)$ with respect to shift. Anti-symmetric $k_2(a)$ (A39) is the negative derivative of $k_1(a)$ with a factor $c_{11}/c_{22}$ (A4) that compensates for regressor norm. Anti-symmetry persists in $\beta _2(a,\beta ,L_2)$ (A7), Fig. 1c; negative value before the zero crossing indicates shift to later time.

Unless $\beta \,{=}\,\mathrm{0}$, the deterministic residual functions $\epsilon (t,a,\beta )$, Fig. 1g, and deterministic residual power $E(a,\beta )$ (A1) as the time integral of $\epsilon ^2(t,a,\beta )$, Fig. 1d, do not vanish for nonzero shift. Under noise, deterministic residual power adds onto stochastic residual power (A16–A17) and via residual variance (A18) leads to over-estimated parameter variance. Despite the different shapes of residual functions for the same amount of shift to earlier and later time, residual power is symmetrical with respect to zero shift.

Higher-order derivatives or time-shifted regressors reduce $\epsilon (t,a,\beta )$ and $E(a,\beta )$, but even an infinite number of derivatives would not accomplish shift to earlier time, supplementary Fig. S1. Fortunately, there is no need for a residual function to vanish, if there is a means to retrieve latency from the coefficients to HRF and time derivative. Magnitude can then be estimated in a model without a time derivative, when the HRF regressor is shifted by the retrieved latency.

Such a means is the former coefficient ratio–latency curve^15^, now with an assignment to the known initial shift as in Liao et al.^23^. That function $a(r,L_1,L_2)$, Fig. 1f, is the part of the ratio function

6 $$\begin{aligned} r(a,L_1,L_2)= & {} \frac{\beta _2(a,\beta ,L_2)}{\beta _1(a,\beta ,L_1)} \end{aligned}$$

(A8), Fig. 1e, between the zero crossings of $\beta _1(a,\beta ,L_1)$ to both sides of $a\,{=}\,\mathrm{0}$, resolved for shift. The overall range of $a(r,L_1,L_2)$ is set by the zero crossings of $\beta _1(a,\beta ,L_1)$, slope is given by $L_i$ ratio (A9), and the linear range extends to shifts beyond 1 s, Fig. 1f. Limit latencies are ±7.27 s for a decomposition into HRF and time derivative, and ±6.41 s for a decomposition into HRF, time derivative, and the constant function.

For an accurate mapping the ratio–latency curve would be calibrated on the regression model specific to the experimental design. Compression of $a(r,L_1,L_2)$ at inclusion of the constant function is a result of correlation with the HRF by which the range of $\beta _1(a,\beta ,L_1)$ becomes larger and the points of zero crossing move towards zero. The coefficients $\beta _i(a,\beta ,L_i)$ lose symmetry with correlations introduced by temporal filtering or further regressors, and narrow spacing of multiple HRFs in a regressor may not allow for zero crossings in $\beta _1(a,\beta ,L_1)$, Fig. 1b. Then, the range of retrievable latency is the monotonous part of $a(r,L_1,L_2)$, Fig. 1f.

Rather than mapping large true latencies back to a limit latency, the central ratio–latency function would confuse such latencies, since a given coefficient ratio occurs at shifts between and beyond the zero crossings of $\beta _1(a,\beta ,L_1)$ in the full ratio function. The same ratio emerges for a positive latency outside limits (which has a positive derivative coefficient and a negative HRF coefficient) and a negative latency within limits (which has a negative derivative coefficient and a positive HRF coefficient), and vice versa, Fig. 1e. Ignoring the origin of the signs along with the other parts of the ratio function, the central part thus maps true latencies outside limits to latencies within limits; the more distant true latencies appear as the smaller retrieved latencies of opposite sign.

Ratio–latency curves from a single HRF and curves from a specific experimental design may not even coincide at $a\,{=}\,\mathrm{0}$ in case of multiple conditions, correlated regressors, or condition-specific shifts, Fig. 1h. Condition-specific calibration is hardly feasible, as the ratio–latency function $a_m(r_m,L_1,L_2)$ for condition *m* would depend on the unknown shifts and magnitudes in all other conditions.

Like the ratio of two estimated coefficients can retrieve shift, the ratio of an estimated coefficient and the explicit or calibrated function value at retrieved shift can retrieve magnitude,

7 $$\beta = \frac{\beta _i(a,\beta ,L_i)/L_i}{\beta _i(a,1,1)},\,i\,{<}\,n.$$

**Figure 2:**
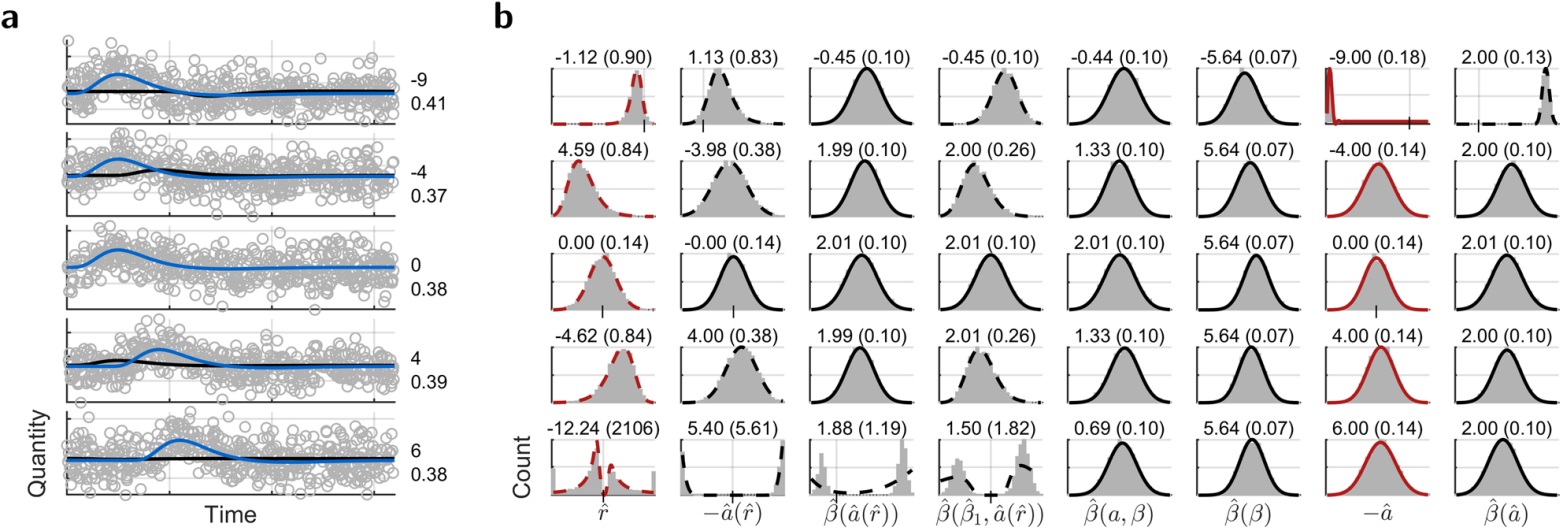
Monte-Carlo simulations for parameters from time derivative and optimization for single HRF. (**a**) Example time courses in time windows of 32 s starting at a negative latency or zero. Black curves are estimates from standard HRF. Numbers indicate true latency in seconds and signal-to-noise ratio, (**b**) Parameter histograms for $\beta \,{=}\,\mathrm{2}$. *Left* to *Right*, (**b1**) Coefficient ratio (6), (**b2**) Latency, (**b3**) Magnitude estimated on shifted HRF regressors with latency from (**b2**), (**b4**) Magnitude by calibration (7) from $i\,{=}\,1$, (**b5**) Enhanced HRF coefficient (A21), (**b6**) Magnitude by power (8), (**b7**) Optimized latency with envelope from the variance relation (9), (**b8**) Magnitude estimated on shifted HRF regressors with latency from (**b7**). Beyond the limits, the signs of enhanced HRF coefficient and magnitude by power are reversed, as they follow the sign change in the HRF coefficient. Outliers in the optimized parameters disappeared when the search for an initial value (see Methods) extended over the true latency. Titles show median and standard deviation. Vertical ticks and grid lines indicate zero. Red envelopes are theoretic, and black envelopes are least-squares fits. Line style indicates the better fit by a normal distribution (solid), or a normal ratio distribution (dashed).

Alternatively, persistence of HRF mean and power (A4) under shift yields

8 $$\beta = \frac{\beta _1(a,\beta ,L_1)}{|\beta _1(a,\beta ,L_1)|}\,\sqrt{s_1\left( E(a,\beta ) + \sum _{i=1}^{n-1} \frac{\beta _i^2(a,\beta ,L_i)}{s_i L_i^2} \right) },$$

adopting the sign from Calhoun et al.^16^. $s_i$ (A12) is the inverse power of the mean-corrected regressor *i*. Equation (8) expands the equation for unnormalized regressors of Steffener et al.^21^ by residual power $E(a,\beta )$ and replaces the norms of plain regressors by those of mean-corrected regressors (A10–A12), so accounts for the constant regressor. Multiplication by $\sqrt{s_1}$ removes a factor common to all voxels. Monte-Carlo simulations reveal that the magnitudes by quotient (7), power (8), and re-estimation are no longer identical in the presence of noise because of different bias or shape of distribution, Fig. 2, as shown next.

### Simulation

Addition of normally distributed noise onto a single shifted HRF in yields normally distributed coefficients with nonzero expectations (A14), hence coefficient ratio ${\hat{r}}(a,L_1,L_2)$ (6) follows a noncentral normal ratio distribution. Within the latency limits of the time derivative, the distribution is left-skewed for positive latency, right-skewed for negative latency, and symmetrical otherwise, Fig. 2b1.

Owing to the central nonlinear coefficient ratio–latency function $a(r,L_1,L_2)$, Fig 1f, latencies are limited and have a less asymmetric distribution. Consistent with the confusion by ambiguous coefficient ratio, Fig. 1e, true latency beyond the limits settles with opposite sign in between. Another confusion is due to noise. Opposite signs and two-peaked distributions emerge at arbitrarily small nonzero noise variance for true latencies within the limits close to the borders. There, the HRF coefficients become small enough to take on values to both sides of zero, while the derivative coefficents retain their large value and sign, Fig. 2b2.

Failure of retrieved latency $-{\hat{a}}$ propagates to the magnitudes by calibration (7) or re-estimation with respective skewed and symmetric distributions. Following the latency confusions above, both distributions include another peak for the sign flip, Fig. 2b3–4.

Successive extension (A13) of magnitude by calibration (7) by further terms of the decomposition (1) arrives at normally distributed magnitude by power (8, A19–A20), same as extension of $\beta _i(a,\beta )/L_i$ (A6). So the distributions from the former extension become successively normal, while those from the latter, among them the enhanced HRF coefficient^16^ (A21), remain normal. By including all terms, magnitude by power has the least variance, smaller than $\beta _i(a,\beta )/L_i$ alone (A15, A20), but gains bias from noise to exceed true magnitude (A14, A19), Fig. 2b5–6.

Smallest variances of magnitude occur at $a\,{=}\,\mathrm{0}$. Elsewhere, re-estimation appears as the best choice in terms of bias and symmetry, Fig. 2b3–5. At sufficient noise variance and extreme latency, optimization may end in outliers if not initialized to a value near the solution, Fig. 2b7–8. At the true HRF latency, the time shift between regressors and voxel time course is zero, deterministic residual power is zero, therefore residual variance is minimal (A16–A17), Fig. 2b8.

Helpful for statistical evaluation, the variance of optimized latency $-{\hat{a}}$ is a multiple of the variance of optimized magnitude ${\hat{\beta }}$,

9 $$\mathrm{var}\bigl (-{\hat{a}}\bigr ) = \frac{\mathrm {var}\bigl ({\hat{\beta }}\bigr )}{{{\hat{\beta }}}^2} \frac{s_2 L_2^2}{s_1 L_1^2}$$

(with the variables as before), relates to the variance of the time derivative coefficient (A22), and provides a proper envelope to the histogram of optimized latency, Fig. 2b7.

### Application

**Figure 3:**
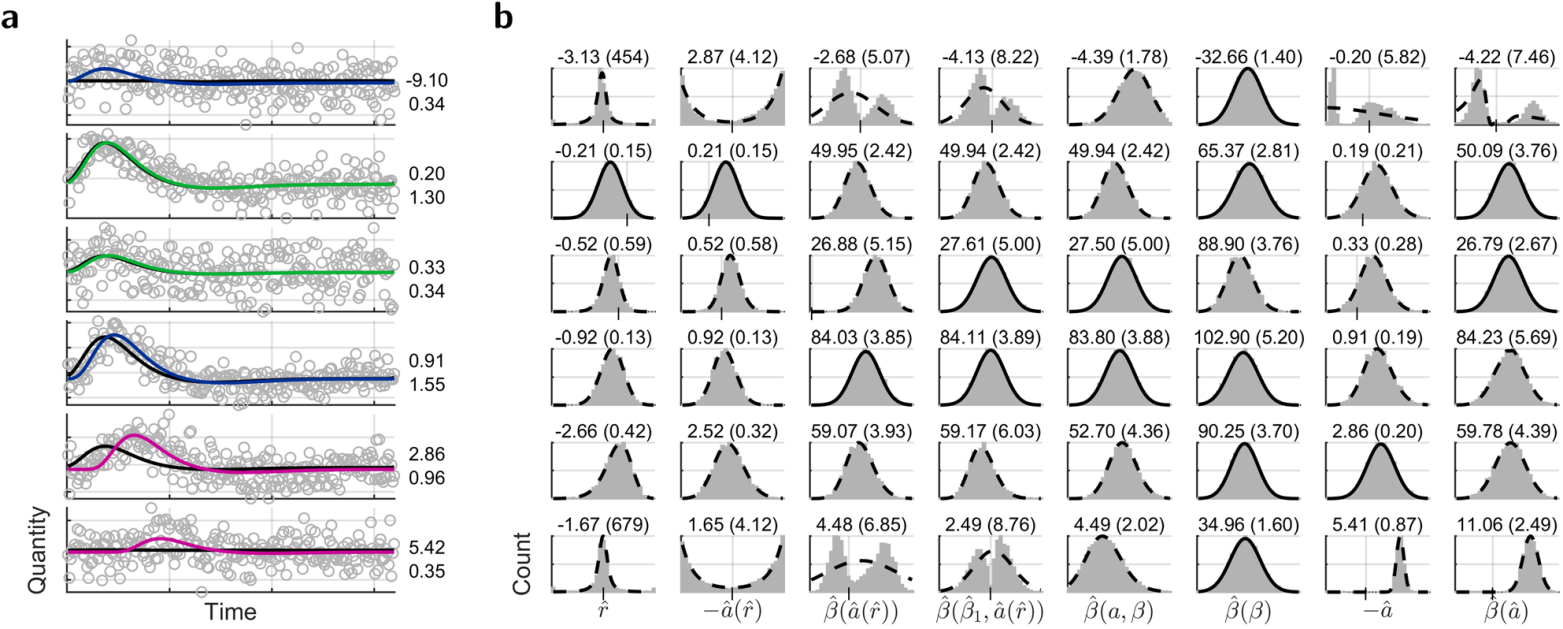
Bootstrapped parameters from fMRI. Time courses come from voxels with best and least t-value in disjunct subsets S1–S3. S1, Significant magnitude, S2, Significant latency and magnitude, S3, Significant latency, magnitude, and magnitude gain over standard HRF. (**a**) Interleaved voxel time course with estimations from standard (black) and optimized HRF (color assignment according to subset, Fig. 4b). Numbers indicate optimized latency in seconds and signal-to-noise ratio, (**b**) Parameter histograms as in Fig. 2. One-peaked distributions for optimized parameters emerged when the search for the initial latency extended over the true latency.

Bootstrapping on residuals^30^, Fig. 3, reproduced the distributions from the Monte-Carlo simulations in Fig. 2, with single-subject fMRI data upon passive viewing in an ideal experimental situation. Long inter-stimulus intervals and restriction to one stimulus modality and one experimental condition (see Methods) should provide clearly separated HRFs and ensure smallest regressor correlation.

With a focus on the immediate impact of time shift on regression coefficients and the smallest reliable latency, the single-subject analysis avoided blurring from averaging over subject group. FMRI data remained in native space, in order to minimize any effect on temporal shape by spatial operation; only the statistical maps were normalized into standard stereotactic space, for assignment of anatomic area.

In the models with a time derivative regressor, latency came from the model-specific coefficient ratio–latency function with limit latencies of ±5.5 s, Fig. 1f D6, that considered the constant regressor and temporal high-pass filtering. Magnitude was estimated voxel-wise in models without a time derivative but with HRFs shifted by retrieved latency, same as with optimization, so different latency alone would have caused different magnitude. Parameters and their differences were assessed at a significance level of $\mathrm{p}\,{<}\,\mathrm{0.05}$ Bonferroni corrected (56225 voxels, $\mathrm{p}\,{<}\,\mathrm{8}{\cdot}\mathrm{10}^{-\mathrm{7}}$ uncorrected) unless stated otherwise. Magnitude gain was defined as the positive difference between the positive magnitudes from optimized and standard HRF, and the t-value for latency made use of the variance relation (9).

**Figure 4:**
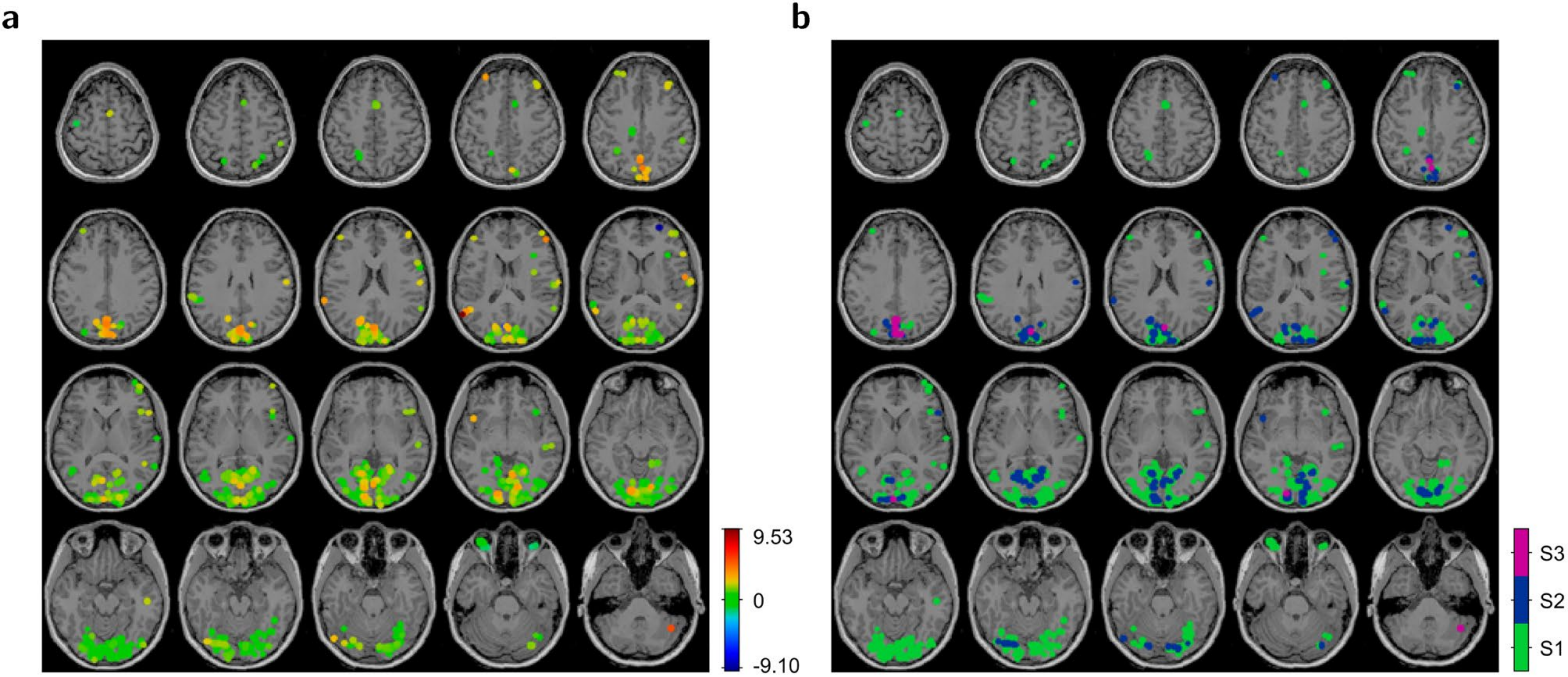
Optimized latency in a native brain. (**a**) Latency map for voxels from the subsets S1–S3 as defined in Fig. 3. The color bar shows latency in seconds, (**b**) Subset map. Pixels were dilated by a disk with a radius of 4 mm, for better visibility.

Optimization revealed significant magnitude gain with latencies close to 3 s in right precuneus in a cluster of six voxels (142 mm$^\mathrm {3}$; peak-voxel MNI coordinates [1.5, −63, 40.5] mm, Brodmann area (BA) 7, five-sigma ($\mathrm{p}\,{<}\,\mathrm{10}^{-\mathrm{7}}$) confidence interval [2.52, 4.83] s), Fig. 4. Significant latency was rarely accompanied by a significant gain in magnitude, and no such gain occurred without a significant latency. Other voxels had an insignificant latency or an insignificant magnitude gain, specifically in the primary (BA 17, V1), secondary (BA 18, V2), and associative (BA 19, V3–V5) visual cortices, or fusiform gyrus (BA 37). These areas contained 77% of all voxels with significant magnitude. No such voxels belonged to primary motor (BA 4), primary auditory (BA 41), or secondary auditory (BA 42) cortices. Similar maps emerged from spatially smoothed data, Fig. S2.

Given the impact on peak and mass centers, Fig. S4, latency was smaller by about 0.5 s when the nonzero stimulus duration was considered by a block response^31^ (A32–A33). Maps therefore contained less voxels that had a significant latency or a significant gain in magnitude, Fig. S3. More voxels had a higher significant latency with impeded perception, Fig. S5.

Among the voxels with significant optimized magnitude at $\mathrm{p}\,{<}\,\mathrm{0.001}$ uncorrected, significant optimized latency had time derivative counterparts of any latency and often was beyond the limits of the time derivative. Only few voxels with different latency persisted at $\mathrm{p}\,{<}\,\mathrm{0.05}$ Bonferroni corrected, Fig. S6. All significant differences in magnitude were due to opposite sign and confined to voxels with significant latency that lacked a significant magnitude gain. Apart from few exceptions that all had optimized latencies beyond the limits (and thus could give rise to large difference to another value of the same sign), the respective latencies had opposite sign, too. Also, the absolute optimized latency was between 3.92 s and 9.68 s, close to a limit and beyond. Taking the optimized latencies for the true latencies, this may support the confusions of latency by coefficient ratio, Fig. 1e, or noise, Fig. 2b2. The connection to negative magnitude is straightforward. Having the wrong sign, the retrieved latency is even further away from the true latency than the standard latency of zero. Hence at re-estimation the true latency remains outside limits where the HRF coefficient is negative, Fig. 1b.

**Figure 5:**
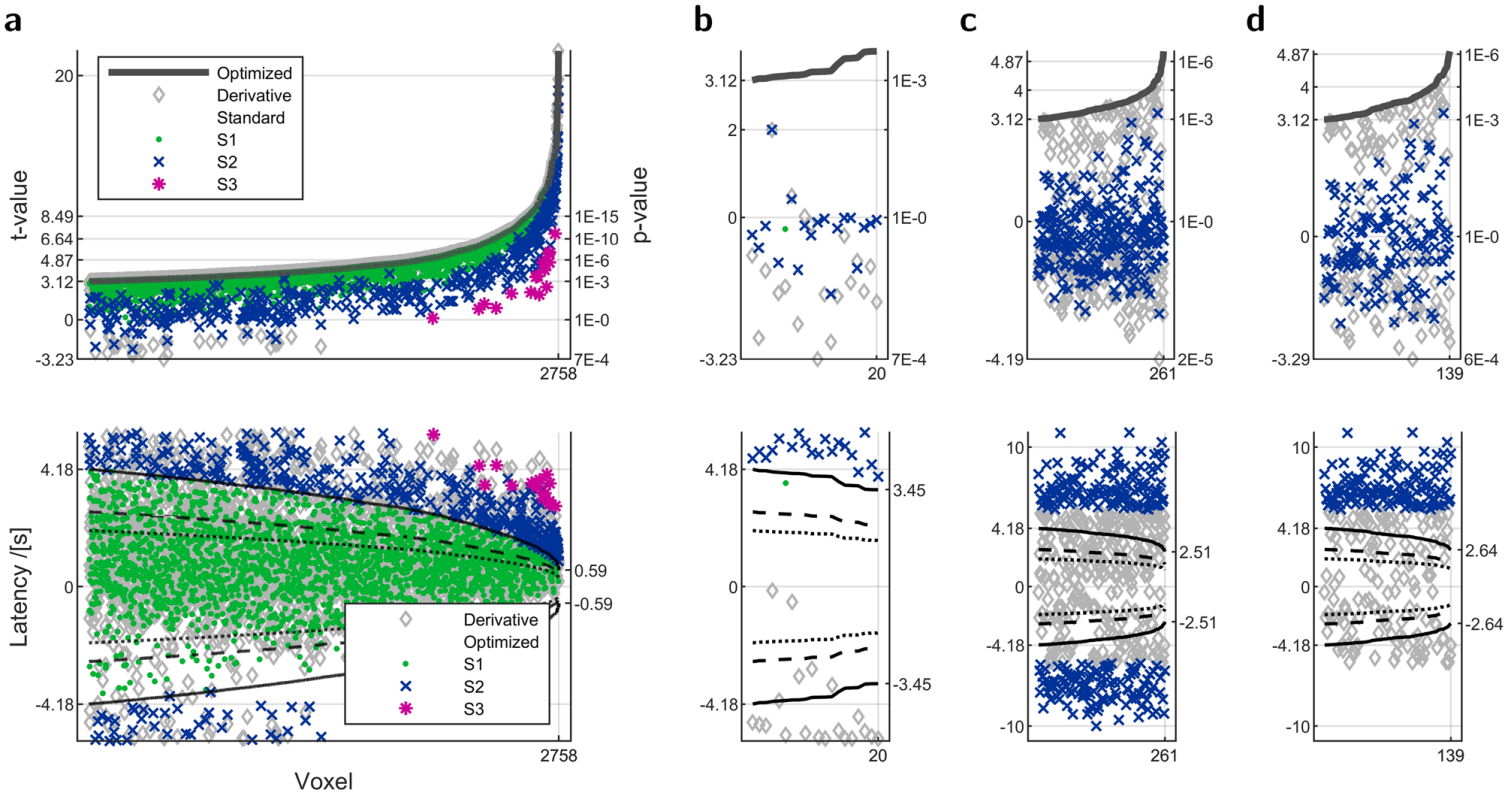
Confusions of latency. Latencies close to the limits of the central ratio–latency function and beyond appear as within-limit latencies of opposite sign. The plots show voxels with optimized magnitude at significance level $\mathrm{p}\,{<}\,\mathrm{0.001}$ uncorrected, sorted by ascending t-value. *Top*, t-value of magnitude, *Bottom*, Latency. (**a**) Optimized latency within ±5.5 s, (**b**) Subset of (**a**), voxels with positive latency and sign flip, (**c**) Optimized latency outside limits, (**d**) Subset of (**c**), voxels with positive latency. In the plots of t-value, markers for the subsets S1–S3 from Fig. 3 are attached to data points from standard HRF. In the plots of latency, they are attached to data points from optimization. Pairs of black lines connect the threshold latencies for significance at $\mathrm{p}\,{<}\,\mathrm{0.05}$ Bonferroni corrected (solid), $\mathrm{p}\,{<}\,\mathrm{0.001}$ uncorrected (dashed), and $\mathrm{p}\,{<}\,\mathrm{0.01}$ uncorrected (dotted). Close ordinate values show lowest (right) and highest (left) thresholds at Bonferroni corrected $\mathrm{p}\,{<}\,\mathrm{0.05}.$ There, an absolute latency must have been larger than 0.59 s (0.37 s at $\mathrm{p}\,{<}\,\mathrm{0.001}$ uncorrected, 0.28 s at $\mathrm{p}\,{<}\,\mathrm{0.01}$ uncorrected).

A lower limit of significant latency is obtained from the voxel with smallest residual power, considering from (9) that the t-value for latency is a multiple of the t-value for magnitude, then resolving the t-threshold of the specific significance level for latency. From that, significant absolute latencies at $\mathrm{p}\,{<}\,\mathrm{0.05}$ Bonferroni corrected could not be smaller than 0.59 s, Fig. 5.

**Figure 6:**
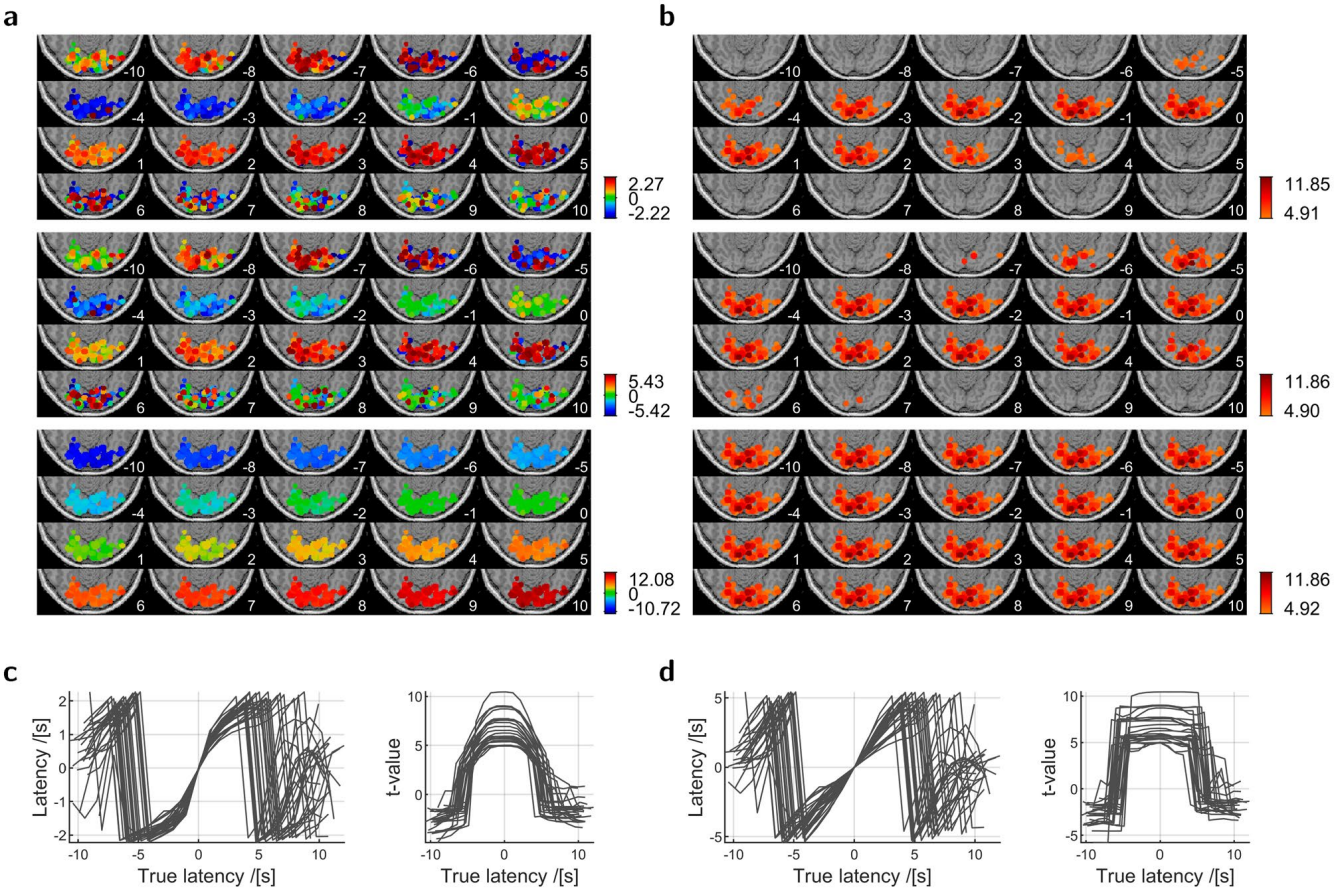
Retrieval of known response latencies. Modeled stimulus onset times were shifted within [−10, 10] s in steps of 1 s in the voxels of the bottom left slice of Fig. 4 that had insignificant optimized latency between −0.72 and 2.08 s and signal-to-noise ratio between 0.34 and 0.84. Negative onset shift moved the modelled HRF to earlier time, thus introduced an additional positive response latency in a voxel time course. Because latency is a difference between true and modeled responses, it persists in another frame where the onset is left untouched and the voxel time course is shifted to later time. Numbers indicate introduced shift in seconds. (**a**) Maps of retrieved latency. *Top*, Time derivative with ratio–latency function from distorted HRF^15^ with latency limits of ±2.2 s, *Middle*, Time derivative with ratio–latency function from shifted HRF, Fig. 1f D6, with limits of ±5.5 s, *Bottom*, Optimization, (**b**) Maps of t-value of estimated magnitude above a significance level of $\mathrm{p}\,{<}\,\mathrm{0.05}$ Bonferroni corrected, (**c**) Latency and t-value as functions of known latency in 30 randomly selected voxels, using the ratio–latency function from distorted HRF. Retrieved latencies are squeezed versions of the true latencies; a known latency of 5 s is retrieved as a latency of 2 s, (**d**) As (**c**), using the ratio–latency function from shifted HRF.

The time derivative approach retrieved known induced latencies within the limits of the ratio–latency function. Because of the latency confusions and associated negative magnitudes, Fig. 5, the latencies changed drastically at the limits and beyond, and the statistical maps of magnitude thinned out. The decomposition (1) takes place irrespective of a ratio–latency function which for a given ratio returns what it has been assigned to at calibration. Systematically squeezed latencies therefore resulted from distorted HRF^15^ while the induced latencies resulted from shifted HRF, Fig. 6. The squeezing reflects the functional relation between true shift and maximum position in distorted HRF.

## Discussion

The decomposition of a shifted HRF into standard HRF and time derivative does not fulfill an addition theorem^32^ as do trigonometric functions, instead for nonzero time shift leaves a nonzero deterministic residual function which adds onto residuals from noise and introduces positive bias into residual power, then leads to overestimated residual and coefficient variances. Voxels with nonzero latencies thus become penalized twice in their t-value, by smaller absolute coefficient in the nominator and larger variance in the denominator.

The residual function explains why the function space spanned by the HRF and derivative basis functions does not include undistorted shifted HRFs, as these would require the deterministic residual function as another basis function. Since this is a function of unknown time shift, knowing the extra function means knowing the result of HRF reconstruction. Further time derivatives would not help, Fig. S1, but reduce the robustness of the estimates.

The HRF decomposition has symmetric and anti-symmetric coefficient functions, therefore has an anti-symmetric coefficient ratio–latency function, Fig. 1f. In contrast to Henson et al.^15^ but same as in Liao et al.^23^, the same displacement to earlier and later time thus results in the same absolute ratio. Calibrations of ratio–latency transfer on regressors of usual experimental designs may have no part in common with the calibration from single HRF which then would return wrong latency. Regressor correlation as one source of deviation^15^ can be minimized by outsourcing correlated regressors to a preceding regression and working on residuals^19^, at a potential cost of destroying measured HRFs.

Smaller range of latency as well as asymmetry in Henson et al.^15^ are due to a definition of latency based on the maximum position in distorted HRF. Asymmetry also comes from a large step size in finite difference approximation; degenerated time derivatives remain distorted and correlated with the constant regressor after orthogonalization to the HRF. The smaller step size used with the dispersion derivative in the SPM software could produce sufficiently orthogonal regressors and make unnecessary an investigation into variants of orthogonalization^24^, to either the HRF regressor alone, or to HRF and constant regressors.

Shape distortion has been misleading and is not an issue with the time derivative. Instead, latency retrieval is limited by experimental design and noise. Limit latencies are given by the zero crossings of the HRF coefficient function or by the monotoneous section of the central ratio–latency function when the zero crossings have disappeared because of small inter-stimulus time. Noise may induce a sign flip when latencies approach the limits, and ambiguous coefficient ratio would map latencies beyond limits to small latencies of opposite sign within limits. Restriction to small latencies thus may eliminate correct latencies and protect wrong ones. Use of the signs of HRF and derivative coefficients together with the full ratio function can avoid part of the confusions but must preclude deactivation. When by sign flip the HRF moves into the wrong direction, the magnitudes by re-estimation will become negative and disappear from maps of brain activation.

In the HRF decomposition, Gamma PDFs of time within the regressors reappear as functions of shift in the correlation integrals and subsequently in the coefficient functions, along with their mutual relations. Apart from a factor, the coefficient functions to singular vectors^23^ arise from time-shifted coefficient functions, by the same linear combinations that produce the singular vectors from time-shifted HRFs. The full set of these vectors can retrieve latencies from within the covered time interval, Fig. S1.

Immediate transfer of regression coefficients into magnitude must consider their respective regressors^16^. Same as distance and velocity, samples of HRF and derivative are different physical quantities with their own units of measure. Because they must compensate towards identical unit in all terms of the decomposition, the HRF and derivative coefficients have different units of measure. The improper addition of values with different units alone can explain the implausible enhanced and reduced magnitudes for the same amount of displacement to later and earlier time^24^.

Enhancement of HRF coefficients^16, 21^ is grounded in preserved signal power under shift but requires norms of mean-corrected regressors which would not additionally underestimate the magnitudes; inclusion of residual power overestimates them. Another sign definition by HRF and derivative coefficients^33^ would be effective only in parts of latencies beyond the limits and produce a wrong positive sign at all shifts to later time when true magnitude is negative. Distributions of magnitude by calibration are skewed, therefore not assessable by statistical tests that rely on normal distributions.

Evidence for region-specific HRF delay from task fMRI remains vague, as long as the neuronal event that underlies a measured BOLD response remains unspecified, and when reaction time is involved. When accepting functional segregation, a BOLD response in a motor area must have come from the requested subject motor response, rather than a preceding stimulation in another modality. Temporal order of BOLD response alone, Mohamed et al.^34^ opposed to Kruggel and von Cramon^28^, would not proof region-specific HRF delay.

As with regression coefficients, a single retrieved latency is meaningless without a statistical evaluation. A minimum significant absolute latency of about 0.6 s from the variance relation exceeds usual slice time differences between neighboring slices which therefore cannot be detected. The observed longer latencies for impeded perception, alternatively the shorter latencies for repetition, require further substantiation.

For shallow minima of residual power as a function of time shift, even a significant latency would not yield a significant gain in magnitude as an indicator of a more exactly reconstructed response. Motor or auditory brain areas did not respond in the passive viewing experiment, and no magnitude gain occurred in visual areas. However, it occurred in the precuneus which is involved in recollection, memory, integration of information towards perceived gestalt, cue reactivity, mental imagery, episodic memory retrieval, and affective response to pain^35^. While any of these could have been among the subject’s actions or perceptual experiences, including her reported afterimages, the experiment is far from claiming any precuneus involvement in the sole task of passive viewing, investigating just one subject. If large delay outside areas that are served by large draining veins is not generally artifactual^36^, the obtained latency may have come from some self-initiated neural activity after stimulation.

The time derivative approach is complicated by latency confusion. Gain in magnitude at re-estimation with shifted HRFs can resolve confusion and give reason to accept a latency.

## Methods

### Implementation

#### Environment

All computation including figure generation ran under MATLAB^®^ 9.7.0.1216025 (R2019b, The MathWorks Inc., Natick, MA, USA); part of figures came from version 9.11.0.1809720 (R2021b) Update 1. MRI data were processed with SPM12 r7219 (The Wellcome Centre for Human Neuroimaging, UCL Queen Square Institute of Neurology, London, UK) modified for explicit functions and optimization. Calibration of ratio–latency curves, translation of ratio into latency, model re-estimation with voxel-specific latency, and the bootstrap were done with an in-house toolbox hosted under SPM. Parameters of the HRF (2) were $p_1\,{=}\,\mathrm{6}$, $p_2\,{=}\,\mathrm{16}$, $q_1\,{=}\,q_2\,{=}\,\mathrm{1 s}^{-\mathrm{1}}$, and $c\,{=}\,\mathrm{1/6}$^20^.

#### Monte-Carlo simulations

Each histogram in Fig. 2 was based on 5000 noise time courses sampled from a normal distribution. That number is the allowed maximum with the applied tests of composite normality^37^. Noise time courses were overlaid on the same signal, a shifted unfiltered HRF without mean correction and onset at 0 s. Signal-to-noise ratio was the root power ratio of signal and noise time courses. HRFs were sampled within [−12, 44] s in steps of 0.02 s. Parameter distributions were identified as normal or normal ratio by Shapiro-Wilk or Shapiro-Francia tests which rejected the null hypothesis of composite normality at significance level $\mathrm{p}\,{=}\,\mathrm{0.01}$. Envelopes for ratio distribution were computed for uncorrelated noncentral normal ratio^38^.

#### Calibration

According to decomposition (1), calibration stepped through given shifts (within [−20, 20] s, Fig. 1), for each of these built a shifted HRF with $\beta \,{=}\,\mathrm{1}$, then estimated the coefficients to the unshifted regressors, computed the required parameter value, finally assigned that value to the given shift. Inverse functions were interpolated from cubic splines.

#### Optimization

Latency was optimized by unconstrained Nelder-Mead nonlinear minimization (MATLAB function fminsearch) of residual power in a model without a time derivative. Optimization started with an initial latency, built a shifted HRF regressor, estimated the coefficients, computed residual power, then continued with a newly determined latency until minimum residual power, then continued with the next voxel. Intended to mitigate the problem of local minima, the initial value was the discrete latency at least residual power upon shifting HRF regressors with step size 0.1 s within the latency limits from the time derivative, for fair comparison.

#### Bootstrap

Latency from ratio–latency transfer (6) and magnitudes by calibration (7) or power (8) involve nonlinear functions of regression coefficients, therefore their variance cannot be approximated from residual variance and must be bootstrapped. 5000 bootstrap samples were used with the residuals method^30^. One bootstrap sample had as many samples as the time course and contained repeated samples.

#### Regressors

Superimposed explicit functions for HRF (2) or time derivative (A27) replaced convolution in the SPM software. One function was placed at each stimulus onset time and the sum sampled at fMRI repetition time (TR). Impulse response was exchanged for block response (A32–A33) when the model considered the nonzero stimulus duration. Same as high microtime resolution and sufficiently small step size, explicit functions allow for exact timing and avoid regressor correlations from finite difference approximation of time derivatives. As a main advantage not ensured with microtime, explicit functions provide differing function values for arbitrarily small shift differences during optimization. They so avoid an early stop of gradient-based search, when a new tested shift falls into the same time bin, is thus associated with the same function value, and no direction of improvement is detected. For consistency with SPM, the derivative regressors were still orthogonalized to the HRF regressor of the same experimental condition, and division by 1/TR times the theoretical integral over the HRF (A25) scaled the regressors as done by the inverse sum over HRF samples. Since the vector dot product of sampled orthogonal functions is nonzero, orthogonalization took a slight effect on the derivative.

#### Shifting

Time shift applied to the HRF rather than the data. Only the regressors of experimental conditions were shifted, by regressor rebuild, after a shift of the underlying time vector. Alternatively, modification of HRF onset parameter would have cut off the initial part of HRFs when the onset was negative, and interpolation would have produced smaller deformation throughout. Shifts involved both the latency in question and the time of the reference slice from slice-time correction.

### Application

#### Subject

One female 29-year-old healthy subject without metallic or electric implants and no known visual or neurological deficits took part in the experiment for no payment, after giving informed consent. The experiment was approved by the Ethics committee of Ulm University (447/20) and conducted in accordance with the Declaration of Helsinki; it has not been registered in a publicly accessible database.

#### MR imaging

Instructed to look at the screen all the time, and head padded to minimize motion artifact, the subject underwent whole-brain gradient echo echo-planar functional imaging during two series of visual stimulation (TR 2000 ms, echo time 33 ms, flip angle 90°, 32 axial-coronar slices, matrix 90×90 pixels, voxel size 2.44×2.44×4 mm^3^, identical image position and axial-coronar orientation), on Siemens MAGNETOM Prisma (Siemens, Erlangen, Germany), using the standard 64-channel head/neck coil. The subject’s structural T1 image was taken from a previous independent study. No subject response was requested or recorded. The first series was the experimental series. The second series served as an independent visual localizer for the slice with maximum visual activation, only to become the reference for slice-time correction of the experimental series and ensure minimum correction of voxel time course in visually activated voxels. Both series involved 15 repetitions of a visual stimulus within a central disk comprising 9.8 visual degrees on a black background, preceded by 8 s of background display awaiting MR equilibration. Stimuli contained a central white fixation cross. The stimulation part of the experimental series was preceded and followed by another 12 s of background display, providing fMRI volumes for time shift. Stimuli of the localizer series (93 volumes) were a black and white checkerboard reversing at 3.75 Hz, displayed for 4 s. Time between onsets was 12 s. The fixation cross appeared between stimulations. Stimuli of the experimental series (257 volumes) were a static image of random pixel value as black or white, displayed for 1 s. Time between onsets as 32 s plus TR/15 yielded equidistant samples (sampling time TR/15) of the one common HRF from interleaved individual responses^39^, with benefit for delay estimates^27^. No fixation cross appeared between stimulations. While risking loss of eye fixation, this avoided permanent visual stimulation and may have circumvented potential effects from habituation, saturation, or saccades away from the stimulus, in relevant voxels. Presentation (Version 18.1. Build 05.12.15, Neurobehavioral Systems, Inc., Berkeley, CA, USA, https://www.neurobs.com) provided stimuli and collected all timing information, including the times of MR volume triggers. Stimuli appeared on a monitor (NordicNeuroLab AS, Bergen, Norway, https://nordicneurolab.com) and a mirror on the MR head cage. The experimental series had to be repeated because of mirror fogging. The subject reported strong experience of afterimages in all functional series.

#### Preprocessing

SPM default settings applied to all preprocessing except reslicing (trilinear interpolation avoided influence of distant voxels) and realignment (acuity increased to 1). For minimum alteration of time course, volumes of the experimental time series were neither coregistered to any other volume, nor spatially normalized. Time courses were corrected for slice time, in order to exclude it as source of delay. In both series the first four volumes until MR equilibrium were discarded and onsets of stimulation defined with respect to the volume trigger of volume 5. Slice 22 contained the strongest response in the aligned localizer series and served as the reference in slice time correction. The correction assumed equal spacing of slice time and considered the buffer time of 20 ms prior to the next volume scan. The number of microtime bins was set to the TR in milliseconds; temporal acuity was thus 1 ms. After spatial realignment (based on six-parameter rigid body transformation) and reslicing, the brain-masked T1 volume was coregistered to the mean volume of the time series, then normalized to MNI space via segmentation and deformation. Normalization parameters were applied only to statistical maps; anatomic area came from SPM Neuromorphometrics^40^ and MRICroN, https://www.nitrc.org/projects/mricron. The EPI series remained unsmoothed for the main analyses, and was smoothed by a 6 mm Gaussian kernel for a separate analysis. The brain mask was built after two runs of segmentation (the first on T1 volume towards bias corrected volume, the second on bias corrected volume). The sum of tissue probability maps for white matter, gray matter and cerebrospinal fluid passed a threshold of 0.1, and a sequence of one dilation, two erosions, and another dilation per slice, each by a 3×3 matrix of ones as the structuring element, filled the gaps. Spatial normalization worked on separate volumes that contained just one peak voxel, used pullback and trilinear interpolation, and usually delivered more than one voxel in standard space from which the first voxel of maximum value was selected. Voxels per subset (S1–S3) or anatomic area were counted in native space. Native anatomic area came from inversely normalized standard atlases and required prior reslicing of the MRICroN Brodmann atlas into the space of the SPM Neuromorphometrics atlas. Inverse normalization used pullback and nearest-neighbour interpolation.

#### Analysis

Stimuli were modeled by blocks of 4 s with the functional localizer, otherwise by impulses with onsets at the ascending flank. Blocks of 1 s were considered as a variant. No additional regressors were included. Voxel time courses and regressors were highpass-filtered with cutoff time 128 s and the models estimated by ordinary least-squares estimation which avoided computations of shift-dependent weighting matrices. During optimization of autoregressive models, different sets of voxels might have entered the underlying sample covariance matrix and required new hyperparameter estimation for combining variance components with each tested latency. Magnitude was not reconstructed by less computationally demanding calibration (7) or power (8) because of non-normal distribution or bias, Fig. 2. All results came from within a mask of significant positive optimized magnitude; deactivation was not a focus. The voxel subsets were defined on optimized latency and magnitude. One-sided one-sample t-tests considered absolute parameter value or difference, with larger-than-zero value or difference as the alternative hypothesis, and used the small residual variance from optimization (A18). The voxels with significant magnitude gain were a proper subset of the voxels with significant magnitude.

## Supplementary Information

Supplementary Information 1.

Supplementary Information 2.

## Data Availability

The MRI dataset is available from the author upon request.

## Code availability

Code for analysis is available from the author upon request.
