# Supplementary material for: Reconstruction of time-shifted hemodynamic response
Source: Sci Rep. 2022 Oct 19;12:17441. doi: 10.1038/s41598-022-17601-5 (PMC9581965; doi:10.1038/s41598-022-17601-5)
Supplement: Supplementary file 1 — Supplementary Information 1. [file 41598_2022_17601_MOESM1_ESM.pdf]

Reconstruction of time-shifted hemodynamic response
Appendix

Regression *Components* For decomposition (1) with $f(t) = f_1(t)$, $f_n(t) = 1$, $f_1(t) \perp f_2(t)$, $f_2(t) \perp f_n(t)$, and $n = 3$,

$$E(a, \beta) = \int_{-\infty}^{\infty} \epsilon^2(t, a, \beta)\, dt \tag{A1}$$

$$\beta_i(a, \beta, L_i) = \begin{cases} \beta\, L_i \sum_{j=1}^{n} w_{ij}\, k_j(a),\ i < n \\ \beta\, L_n \sum_{j=1}^{n} w_{nj}\, k_j(a) + C\, L_n,\ \text{otherwise} \end{cases} \tag{A2}$$

$$\boldsymbol{W} = \{w_{ij}\} = \frac{1}{1 - r_{1n} r_{n1}} \begin{bmatrix} 1 & 0 & -r_{n1} \\ 0 & 1 - r_{1n} r_{n1} & 0 \\ -r_{1n} & 0 & 1 \end{bmatrix} \tag{A3}$$

$$r_{ij} = c_{ij} / c_{jj}$$

$$c_{ij} = c_{ji} = \int_{-\infty}^{\infty} f_i(t)\, f_j(t)\, dt \tag{A4}$$

$$r_{1n} = k_n(0) = k_n(a)$$

$$z = r_{1n} r_{n1} \tag{A5}$$

$$\beta_1(a, \beta, L_1) = L_1\, \beta\ (k_1(a) - z)\, /\, (1 - z) \tag{A6}$$

$$\beta_2(a, \beta, L_2) = L_2\, \beta\, k_2(a). \tag{A7}$$

Retrieval Owing to (A2) unknown β cancels out, and z (A5) just involves known regressor correlations, so the inverse of coefficient ratio

$$r(a, L_1, L_2) = \frac{\beta_2(a, \beta, L_2)}{\beta_1(a, \beta, L_1)} = \frac{L_2}{L_1} \frac{k_2(a)(1 - z)}{k_1(a) - z} \tag{A8}$$

can deliver shift. From (A40), approximation for small ratio is

$$a \approx \frac{L_1}{L_2}\, r(a, L_1, L_2). \tag{A9}$$

Since $k_2(0) = 0$ (A39), the reverse ratio $\beta_1(a, \beta, L_1) / \beta_2(a, \beta, L_2)$ would suffer from division by zero at $a = 0$.

Equivalence Subtraction of $\beta_n(a)\, f_n(t)$ from both sides of (1) removes the function means (indicated by tilde ~) and the bias C,

$$\begin{aligned} \beta\, \tilde{f}_1(t{+}a) &= \sum_{i=1}^{n-1} \frac{\beta_i(a,\beta,L_i)}{L_i}\, \tilde{f}_i(t) + \varepsilon(t,a,\beta) && \text{(A10)} \\ \tilde{c}_{ij} \;=\; \tilde{c}_{ji} &= \int_{-\infty}^{\infty} \tilde{f}_i(t)\, \tilde{f}_j(t)\, dt && \text{(A11)} \\ \tilde{c}_{ii} &= 1/s_i. && \text{(A12)} \end{aligned}$$

Extension Since $E(a,1) + \sum_{i=1}^{n-1} \beta_i^2(a,1,1)/s_i = 1/s_1$, extension of magnitude by calibration (7) with other terms of (1) except n,

$$\beta \;=\; \sqrt{\frac{E(a,\beta) + \sum_{i=1}^{n-1} \beta_i^2(a,\beta,L_i)/(s_i L_i^2)}{E(a,1) + \sum_{i=1}^{n-1} \beta_i^2(a,1,1)/s_i}}, \qquad \text{(A13)}$$

is equal to the same extension of $\beta_i(a,\beta)/L_i$, and is equal to magnitude by power (8).

Noise *Distributions* With unknown noise variance σ^2 and the degrees of freedom f as the number of samples K minus the rank of the regressor matrix,

$$\begin{aligned} \hat{\beta}_i(a,\beta,L_i) &\sim \mathcal{N}\left(\mu_i, \nu_i\right), \\ \mu_i &= \beta_i(a,\beta,L_i) && \text{(A14)} \\ \nu_i &= \sigma^2 s_i L_i^2 && \text{(A15)} \end{aligned}$$

$$\begin{aligned} \hat{E}(a,\beta) &\sim \mathcal{N}(\mu,\, \nu) \\ \mu &= E(a,\beta) + \sigma^2 f && \text{(A16)} \\ \nu &= 2\sigma^2\big(E(a,\beta) + \mu\big) && \text{(A17)} \\ s_R^2 &= E(a,\beta)/f + \sigma^2 && \text{(A18)} \end{aligned}$$

$$\begin{aligned} \hat{\beta}(\beta) &\sim \mathcal{N}(\mu, \nu) \\ \mu &= \sqrt{\beta^2 + K\sigma^2 s_1} && \text{(A19)} \\ \nu &= \sigma^2 s_1 \left(1 + \beta^2/\mu^2\right)/2 \;\le\; \sigma^2 s_1. && \text{(A20)} \end{aligned}$$

The relations for enhanced HRF coefficient $\hat{\beta}(a,\beta)$ have K replaced by 1 and β^2 replaced by smaller $b^2(a,\beta)$ where

$$b(a,\beta) \;=\; \frac{\beta_1(a,\beta,L_1)}{|\beta_1(a,\beta,L_1)|} \sqrt{s_1 \sum_{i=1}^{n-1} \frac{\beta_i^2(a,\beta,L_i)}{s_i L_i^2}}. \qquad \text{(A21)}$$

Latency variance Ratio distribution has variance as

$$\mathrm{var}(\hat\beta_2/\hat\beta_1) \;=\; E(\hat\beta_2^2)\,E(1/\hat\beta_1^2) - E^2(\hat\beta_2)\,E^2(1/\hat\beta_1),$$

where E is an expected value. At optimum latency $E^2(\hat\beta_2) = 0$ and $\hat\beta = \hat\beta_1$. With $-\hat a \approx \hat\beta_2/\hat\beta_1$, $\mathrm{var}(\hat\beta_2) = E(\hat\beta_2^2)$, $\mathrm{var}(\hat\beta_1) = \sigma^2 s_1 L_1^2$, $\mathrm{var}(\hat\beta_2) = s_2/s_1\, L_2^2/L_1^2\,\mathrm{var}(\hat\beta_1)$, and $E(1/\hat\beta_1^2) = 1/\hat\beta_1^2$ for significant $\hat\beta_1$, this leads to (9) and further to

$$\mathrm{var}\big(-\hat a\big) \;=\; \frac{\sigma^2 s_2 L_2^2}{\hat\beta^2} \;=\; \frac{\nu_2}{\hat\beta^2}. \tag{A22}$$

ν_2 (A15) is the variance of the time derivative coefficient.

HRF *Definition* The HRF $h(t)$ (2) consists of Gamma PDFs of time t,

$$g(t,p,q) \;=\; \frac{d}{dt}F(t,p,q) \;=\; \begin{cases} \frac{q^p\, t^{p-1}\, e^{-qt}}{\Gamma(p)},\ t > 0 \\ 0,\ \text{otherwise} \end{cases} \tag{A23}$$

$$F(t,p,q) \;=\; \begin{cases} 1 - \sum_{k=0}^{p-1} \frac{(qt)^k}{k!}\, e^{-qt},\ t > 0 \\ 0,\ \text{otherwise,} \end{cases} \tag{A24}$$

$p,\ q > 0$. $F(t,p,q)$ is the Gamma cumulative density function, $g(\infty,p,q) = 0$, and $F(\infty,p,q) = 1$. The HRF and the constant regressor are correlated,

$$\int_{-\infty}^{\infty} h(t)\,dt \;=\; F(t,p_1,q_1) - c\,F(t,p_2,q_2)\Big|_0^{\infty} \;=\; 1 - c. \tag{A25}$$

Basis functions Derivatives of Gamma PDFs are linear combinations of Gamma PDFs with different shape parameter,

$$\frac{d^n}{dt^n} g(t,p,q) \;=\; q^n \sum_{k=0}^{\min(n,p-1)} \binom{n}{k} (-1)^{n-k}\, g(t,p{-}k,q), \tag{A26}$$

where $\binom{n}{k}$ is the Binomial coefficient. Then, the HRF has a first derivative with respect to time t as

$$\begin{aligned} \frac{d}{dt}\big(g(t,p_1,q_1) - c\,g(t,p_2,q_2)\big) \;&=\; q_1\,\big(g(t,p_1{-}1,q_1) - g(t,p_1,q_1)\big)\ \ldots \\ &\quad - c\,q_2\,\big(g(t,p_2{-}1,q_2) - g(t,p_2,q_2)\big), \end{aligned} \tag{A27}$$

and with $p_1 = l/d_1$ and $q_1 = 1/d_1$ has a first derivative with respect to dispersion of response d_1 as

$$\frac{d}{dd_1}\, g(t,p_1,q_1) \;=\; q_1^2\,\left(t - \frac{p_1}{q_1}\,\big(1 + \ln(q_1 t) - \psi\,(p_1)\big)\right)\, g\,(t,p_1,q_1)\,, \tag{A28}$$

`http://www.wolframalpha.com`, where ln is the natural logarithm and ψ the Digamma function.

The time derivative is orthogonal to the HRF,

$$\int_{-\infty}^{\infty} h(t)\,\frac{d}{dt}h(t)\,dt \;=\; h^2(t)\,\Big|_0^{\infty} - \int_{-\infty}^{\infty} h(t)\,\frac{d}{dt}h(t), \tag{A29}$$

the dispersion derivative is not. Both have zero mean,

$$\int_{-\infty}^{\infty} \frac{d}{dt}h(t)\,dt \;=\; g(t,p_1,q_1) - c\,g(t,p_2,q_2)\,\Big|_0^{\infty} \;=\; 0 \tag{A30}$$

$$\begin{aligned}\int_{-\infty}^{\infty} \frac{d}{dd_1}\,g(t,p_1,q_1)\,dt \;=\; & q_1 p_1 \Bigg\{ F(t,p_1{+}1,q_1)\,\Big|_0^{\infty} + \ln(q_1) - \psi(p_1)\;\ldots \\ & - \big(1+\ln(q_1)-\psi(p_1)\big)\,F(t,p_1,q_1)\,\Big|_0^{\infty} \Bigg\} \;=\; 0.\end{aligned} \tag{A31}$$

Block response This is a response to block stimuli which are the differences of two Heaviside step functions, one with onset time at the start of stimulation, the other with onset time at the end. Then, since a step function is the time integral of a Dirac impulse and a step response is the time integral of an inpulse response, a block response is the difference between the time integrals of two HRFs with different onset time, and the time derivative of a block response is the difference between two HRFs with different onset time,

$$\begin{aligned} f(t) \;=\; & F(t,p_1,q_1) - c\,F(t,p_2,q_2)\;\ldots \\ & - \big(F(t{-}d,p_1,q_1) - c\,F(t{-}d,p_2,q_2)\big) \end{aligned} \tag{A32}$$

$$\frac{d}{dt}f(t) \;=\; h(t) - h(t{-}d), \tag{A33}$$

using (A23)–(A24). d is the difference between onset times.

Decomposition In (5),

$$S(a,p,q) \;=\; \begin{cases} 1, & a>0, \\ 1 - F(-a,p,q), & \text{otherwise.} \end{cases} \tag{A34}$$

The nth derivative of component $G(a,p_i,p_j)$ is

$$\begin{aligned}\frac{d}{da^n}G(a,p_i,p_j) \;&=\; (-q_j)^n \sum_{k=0}^{\min(n,p_i-1)} \binom{n}{k} (-1)^{n-k}\,G(a,p_i,p_j{-}k) \\ &=\; q_i^n \sum_{k=0}^{\min(n,p_i-1)} \binom{n}{k} (-1)^{n-k}\,G(a,p_i{-}k,p_j).\end{aligned} \tag{A35}$$

The correlation integrals between shifted unshifted HRF and their derivatives (superscript $^{(n)}$ abbreviates $\frac{d}{da^n}$) make use of the equality of HRF derivatives with respect to shift and time, $\frac{d}{da}h(t{+}a) = \frac{d}{dt}h(t{+}a)$,

$$\begin{aligned}\int_{-\infty}^{\infty} h(t{+}a)\, h(t)\, dt &=: H(a)\\ &= G(a,p_1,p_1) - c\, G(a,p_1,p_2)\ \ldots\\ &\qquad - c\Big(G(a,p_2,p_1) - c\, G(a,p_2,p_2)\Big)\end{aligned} \tag{A36}$$

$$\int_{-\infty}^{\infty} h^{(m)}(t{+}a)\, h^{(n)}(t)\, dt = (-1)^n\, H^{(m+n)}(a) \tag{A37}$$

$$k_1(a) = \frac{\int_{-\infty}^{\infty} h(t{+}a)\, h(t)\, dt}{\int_{-\infty}^{\infty} h^2(t)\, dt} = \frac{H(a)}{H(0)} \tag{A38}$$

$$\begin{aligned}k_2(a) &= \frac{\int_{-\infty}^{\infty} h(t{+}a)\, h^{(1)}(t)\, dt}{\int_{-\infty}^{\infty} \left(h^{(1)}(t)\right)^2\, dt} = \frac{H^{(1)}(a)}{H^{(2)}(0)}\\ &= \frac{H(0)}{H^{(2)}(0)}\, \frac{d}{da} k_1(a).\end{aligned} \tag{A39}$$

$H(0) = c_{11}$ and $H^{(2)}(0) = -c_{22}$ (A4). $k_1(0) = 1$ (A38) and $k_2(0) = 0$ (A29), so with (A39)

$$\left.\frac{d}{da}\frac{\beta_2(a,\beta,L_2)}{\beta_1(a,\beta,L_1)}\right|_{a=0} = \left.\frac{L_2}{L_1}\,\frac{d}{da}k_2(a)\right|_{a=0} = \left.\frac{L_2}{L_1}\,\frac{H^{(2)}(a)}{H^{(2)}(0)}\right|_{a=0} = \frac{L_2}{L_1}. \tag{A40}$$
