# Supplementary material for: Reconstruction of time-shifted hemodynamic response
Source: Sci Rep. 2022 Oct 19;12:17441. doi: 10.1038/s41598-022-17601-5 (PMC9581965; doi:10.1038/s41598-022-17601-5)
Supplement: Supplementary file 2 — Supplementary Information 2. [file 41598_2022_17601_MOESM2_ESM.pdf]

Reconstruction of time-shifted hemodynamic response Supplementary information

Analysis

With parameters as in the Methods, all HRF derivatives go back to the same finite set of 16 Gamma PDFs. The regression coefficients to HRF derivatives are linear combinations of regression coefficients to the Gamma PDFs, and the same latter coefficients emerge from a Taylor series, by decomposing each derivative into its Gamma PDF parts and summing over all coefficients of each PDF. The higher-order derivatives are not all mutually orthogonal.

The full basis set of Gamma PDFs can accomplish shift to later but not to earlier time, while a basis system of HRFs with different time shift (or the full set of their mutually orthogonal singular vectors [23], see the reference in the article) can do so within the covered interval, **Figure S1**.

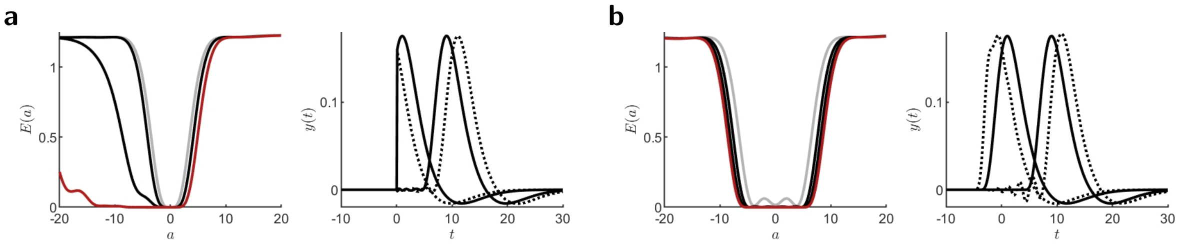

Figure S1: Performance of basis sets. *Left*, Broadening of the zero range of residual power $E(a)$ from a successively larger number of functions (smallest in gray, largest in red), *Right*, Limit functions $y(t)$ for shifts of ± 4 s (solid) and ± 6 s (dotted). Time t and shift a are in seconds. (**a**) Regression on orthogonalized HRF derivatives. Curves of residual power comprise 1, 2, 6, and 15 derivatives, (**b**) Regression on singular vectors of HRFs shifted within $[-5, 5]$ s, with step size 1 s. Curves of residual power comprise the first three to all eleven singular vectors. Compared to HRF and time derivative, the first two singular vectors (not shown) come with a larger range of the ratio–latency function [21] and a broader but centrally upwarped residual power function; the warping is more prominent at wider intervals and suppressed by further singular vectors. Symmetry presupposes both symmetric intervals with respect to $a = 0$ and equal spacing of the shifted HRFs.

Application

No qualitative change of a map came from spatial smoothing, **Figure S2**.

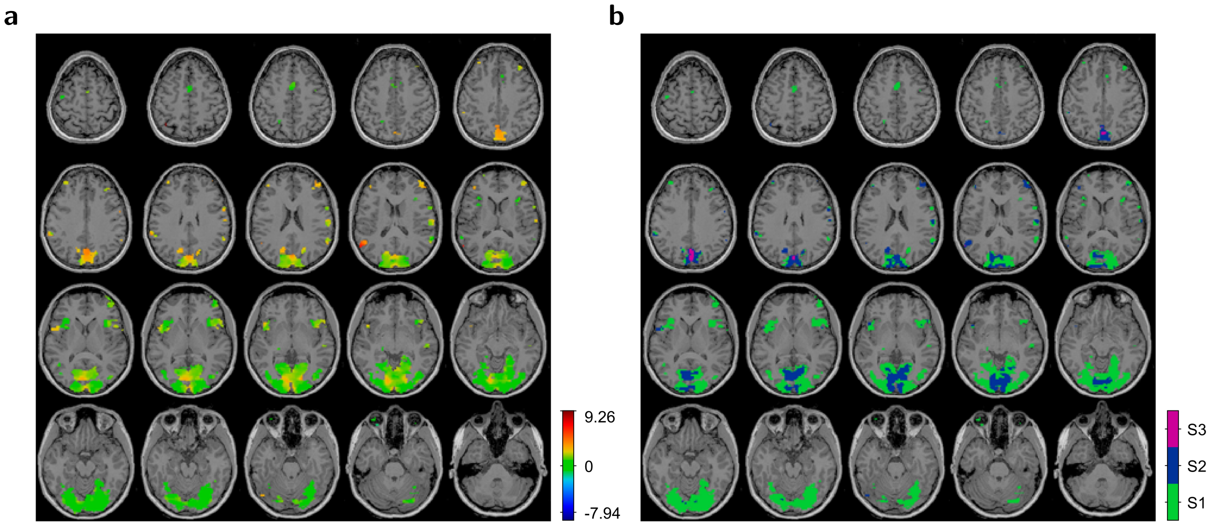

Figure S2: As **Figure 4**, spatial smoothing with a 6 mm isotropic Gaussian kernel. Pixels were not dilated.

Less voxels had a significant latency or a significant gain in magnitude when the stimulus duration was considered by a block response, **Figure S3**. Compared to impulse, peaks and mass centers of responses to blocks of 1 s occur about 0.5 s later, **Figure S4**.

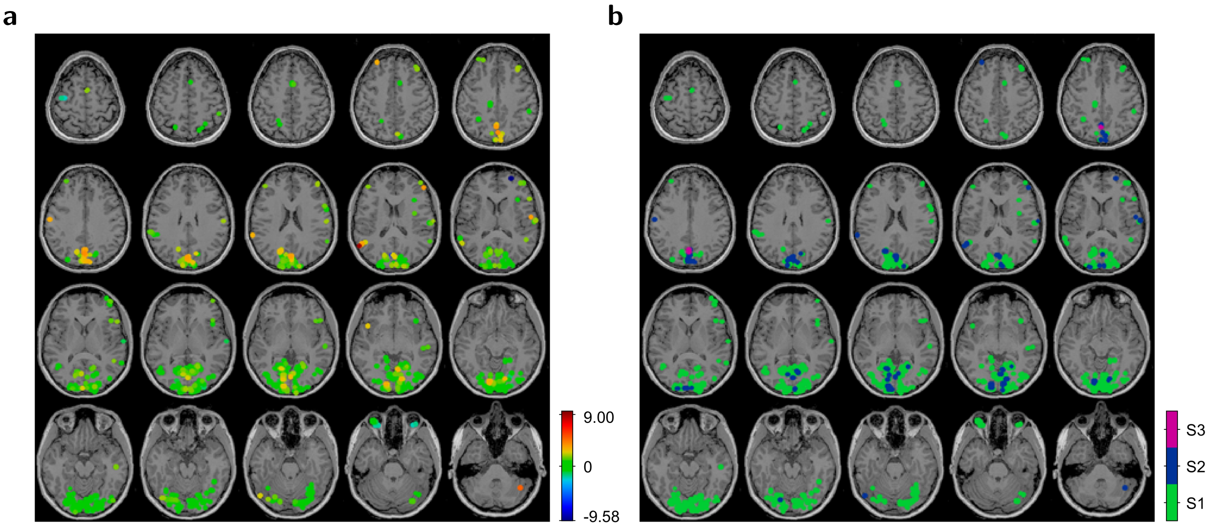

Figure S3: As **Figure 4**, stimulus duration 1 s.

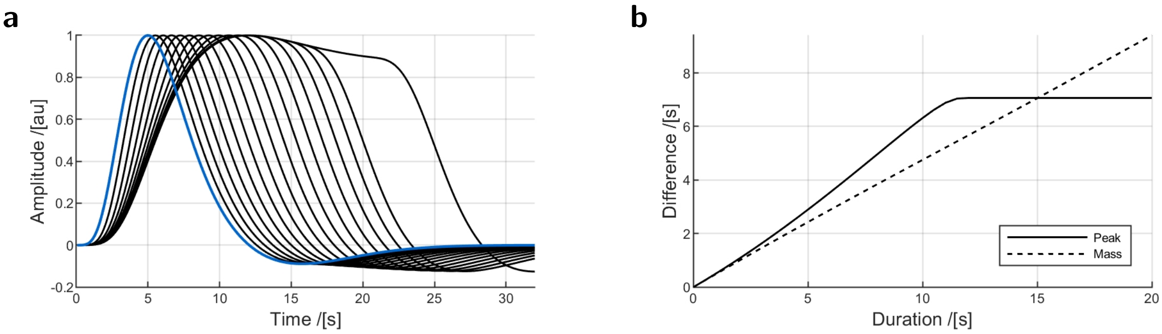

Figure S4: Effect of stimulus duration on latency. (**a**) Responses for different durations as in Henson & Friston [31] FIGURE 14.2 b, scaled to the same maximum. The HRF is shown in cyan, (**b**) Peak and center-of-mass difference with respect to the HRF as a function of duration. The center of mass was based on the positive part of the response.

More voxels had a higher significant latency with impeded perception. The increase in latency was significant, **Figure S5**.

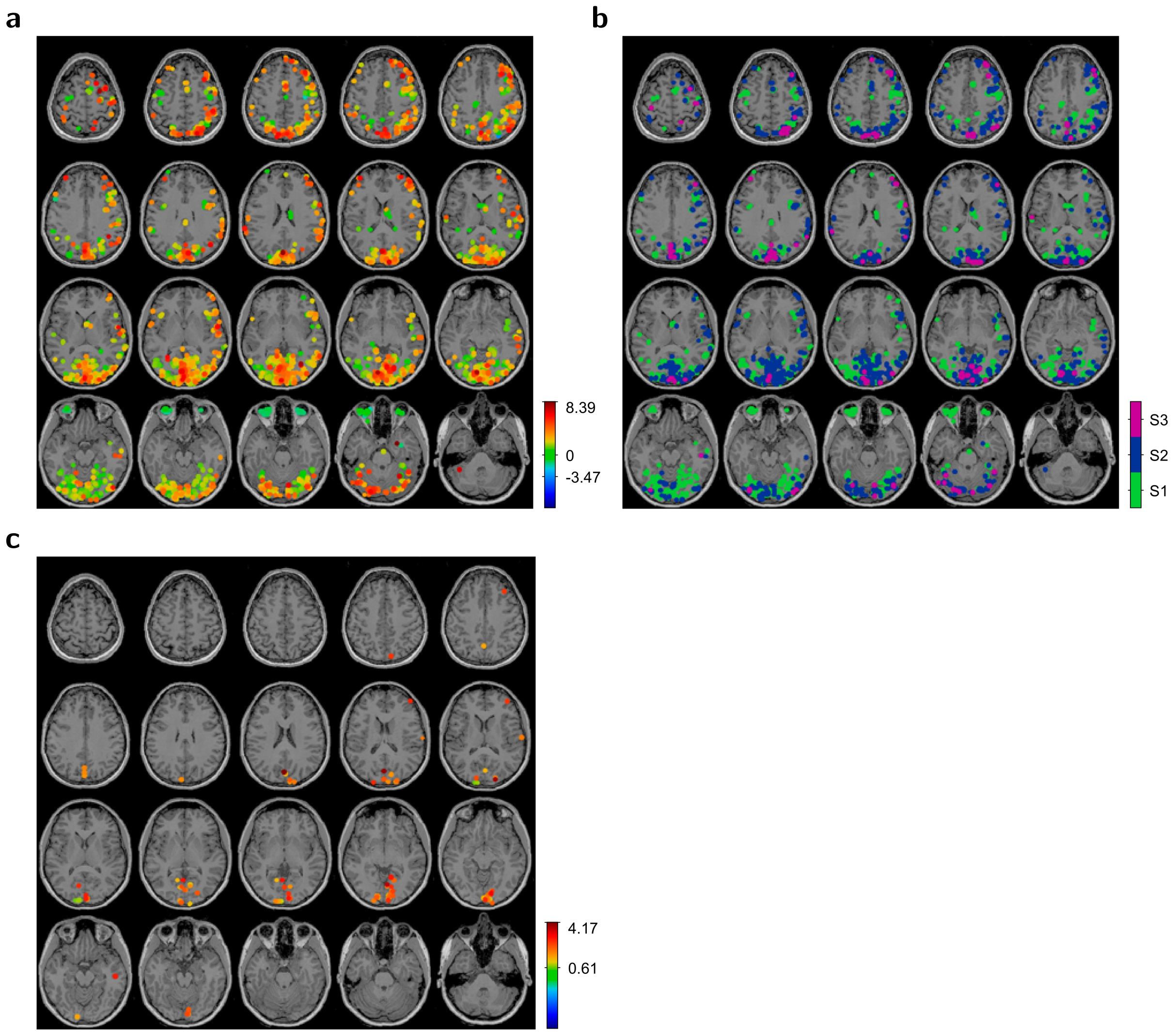

Figure S5: Maps from impeded perception. (**a**,**b**) As **Figure 4a,b**, (**c**) Significantly longer latency within the intersection of voxels with significant magnitude in normal and impeded perception at $p < 10^{-7}$ uncorrected, **Figure 4a** and **Figure S5a**, after coregistration of first volumes, taking latency and mask volumes in tow. Reslicing by nearest-neighbour interpolation prevented the combination of strongly differing latencies from the neighborhood. The color bar indicates latency difference in seconds.

Voxels with statistically identical latency from time derivative and optimization were part of all subsets S1–S3. Differences occured in subset S2, **Figure S6**.

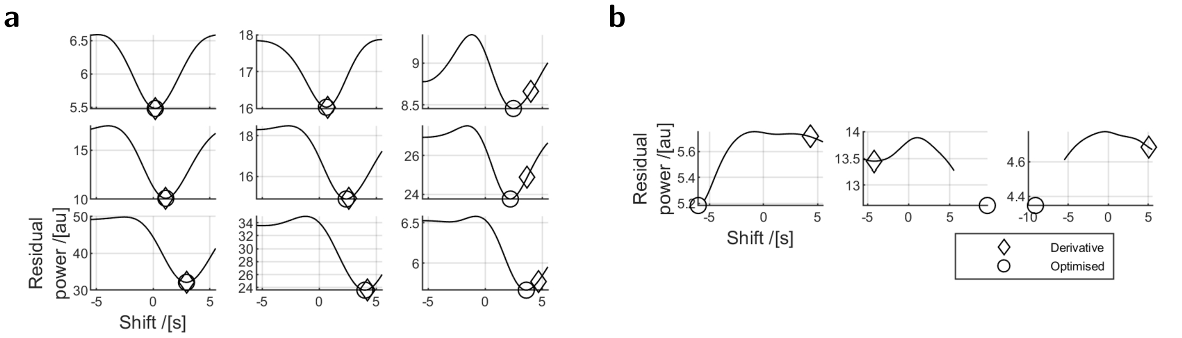

Figure S6: Residual power as a function of shift in selected voxels. *Left*, Least, *Middle*, Median, *Right*, Maximum latency difference between time derivative (diamond) and optimization (circle) approaches. *Top* to *Bottom*, Subsets S1–S3. (**a**) Identical latency, (**b**) Different latency.
